# Dietary Full-Fat Rice Bran or Glucose Regulates Bile Acid Circulation, Colonic Microbiota, and Short-Chain Fatty Acids in Pigs During Chronic Cold Stress

**DOI:** 10.3390/ani15223232

**Published:** 2025-11-07

**Authors:** Yusong Zheng, Yang Zhao, Ze Wang, Guodong Sun, Teng Teng, Baoming Shi

**Affiliations:** College of Animal Science and Technology, Northeast Agricultural University, Harbin 150030, China; s230502021@neau.edu.cn (Y.Z.); zhaoyang0921777@163.com (Y.Z.); wangze20001023@126.com (Z.W.); s976031645@163.com (G.S.)

**Keywords:** full-fat rice bran, glucose, chronic cold stress, bile acid enterohepatic gut microbiota, SCFAs

## Abstract

**Simple Summary:**

Chronic winter cold stress challenges animal husbandry in cold regions, with insufficient precise dietary adjustments for animals’ specific nutritional needs. To address this, 18 Yorkshire pigs were randomly divided into three groups (basal diet, full-fat rice bran diet, glucose diet) for a 22-day cold exposure experiment, to assess the two diets’ effects on pigs’ growth performance, antioxidant capacity, gut microbiota, and bile acid circulation. Key results: The full-fat rice bran diet reduced cold-exposed pigs’ average daily feed intake, increased plasma superoxide dismutase activity, and lowered colonic Rikenellaceae_RC9_gut_group and *Campylobacter* abundance. The glucose diet improved growth performance and decreased colonic Prevotellaceae_NK3B31_group abundance. Both diets reduced colonic *Alloprevotella*, *Bradymonadales*, and *Erysipelotrichaceae* abundance, promoted short-chain fatty acid production, activated the Farnesoid X receptor signaling pathway, and increased fecal bile acid excretion. In conclusion, dietary full-fat rice bran or glucose regulates growth performance, antioxidant capacity, bile acid circulation, and gut microbiota in chronically cold-stressed pigs, providing a new basis for resolving cold-induced adverse effects via nutritional regulation.

**Abstract:**

Chronic cold stress is a severe test that animals in cold regions have to face during winter. However, the lack of precise dietary adjustments for animals in cold regions represents a significant gap in addressing their nutritional needs. Full-fat rice bran is one of the feed materials rich in protein, lipids, dietary fiber, and antioxidant-active substances. Glucose is the most common quick energy supply. We determined dietary full-fat rice bran and glucose can improve the growth and health of cold-exposed pigs. A total of 18 Yorkshire pigs were randomized to one of three treatment groups (basal diet, full-fat rice bran diet (20%), and glucose diet (10%)) for 22 d to evaluate the effects of full-fat rice bran and glucose on growth performance, antioxidants, microbiomes, and bile acid circulation in cold-exposed pigs. The results showed that dietary full-fat rice bran decreased the average daily feed intake (*p* < 0.05), increased superoxide dismutase (SOD) activity in plasma (*p* < 0.05), and decreased *Rikenellaceae_RC9_gut_group* and *Campylobacter* in the colon microbiota of cold-exposed pigs (*p* < 0.05). Dietary glucose improved the growth performance of cold-exposed pigs and decreased the abundance of *Prevotellaceae_NK3B31_group* (*p* < 0.05) in the colon microbiota of cold-exposed pigs. Dietary full-fat rice bran and glucose both downregulated the abundance of *Alloprevotella*, *Bradymonadales*, and *Erysipelotrichaceae* in colon microbiota (*p* < 0.05), promoted the production of SCFAs, and activated the FXR-CYP7A1 signaling pathway. Dietary full-fat rice bran or glucose promoted fecal excretion of bile acids. This study demonstrated that dietary supplementation with full-fat rice bran or glucose can improve the colonic microbiota structure and SCFA levels in cold-exposed pigs. When comparing the two dietary strategies, the glucose-supplemented diet is more beneficial to the growth performance of cold-exposed pigs, while the full-fat-rice-bran-supplemented diet is more conducive to enhancing the function of their antioxidant system. Additionally, dietary supplementation with full-fat rice bran or glucose can also regulate the bile acid circulation in pigs, thereby enhancing their cold adaptation ability.

## 1. Introduction

Humans and animals in high-latitude regions often experience long autumn and winter seasons. In the depths of winter, ambient temperatures in these areas reach even −30 °C. The animals on small and medium-sized farms suffer from cold stress due to inadequate warmth and poor rearing conditions. At present, the existing conclusions indicate that chronic cold stress has negative impacts on growth performance, antioxidant defense systems, inflammation, endocrines, and metabolism of animals [1,2,3,4,5]. Conventional dietary material selection fails to adequately address the nutritional needs of animals under cold stress, thereby heightening the risks and costs of high-latitude animal husbandry [6]. The main strategies for alleviating cold stress have focused on heat production in brown adipose tissue and insulin sensitivity in mammals during cold exposure [7,8,9]. However, there is an urgent need to identify a feasible dietary nutrition strategy to improve the growth and health status of cold-exposed pigs.

Rice bran, one of the main by-products of rice, is considered as a valuable animal feed material. Full-fat rice bran is rich in protein, fats, and dietary fiber [10]. Remarkably, the proteins in rice bran are hypoallergenic, which can be used as an ingredient in foods [11,12]. In recent years, several active compounds, such as tocopherol, tocotrienol, ferulic acid, phenolic compounds, and nucleotides, have been identified in rice bran [13,14,15]. These antioxidants may contribute to the animal’s health. Prior research has demonstrated the ameliorative effects of full-fat rice bran on lipid peroxidation and oxidative stress in porcine hearts induced by prolonged cold stimulation [16]. In addition, the dietary fiber in rice bran is mainly arabinoxylan, a kind of hemicellulose with anti-inflammatory and antioxidant properties [17]. There has been evidence that dietary rice bran protects animals from intestinal pathogens and diarrheal diseases to a certain extent, and regulates the microbiota [18,19]. Glucose supplementation ameliorated the disturbance of small intestinal AA transport and the destruction of muscle AA pools (mainly BCAAs) induced by chronic cold exposure in poorly cold-acclimated Yorkshire pigs [20]. Dietary glucose supplementation could reduce the activation of the TLR4 pathway induced by cold stress [2]. Dietary glucose supplementation can alleviate gut microbiota dysbiosis and intestinal barrier damage caused by cold stress [21]. Therefore, it is worth researching whether dietary full-fat rice bran can regulate the antioxidant capacity and gut microbiota of pigs during chronic cold stress.

When pigs are exposed to low temperatures for extended periods, their energy expenditure increases. Carbohydrates are the primary source of energy for growing pigs. Carbohydrates in the diet are converted into glucose by digestive enzymes in the gut lumen [22]. Glucose is a flux substance that maintains energy balance in cells, and meets energy requirements through the glycolysis pathway [23]. Excessive glucose consumption during cold stress triggers the upregulation of lipolysis and fatty acid oxidation to meet energy demands. Notably, this upregulation detrimentally impacts the gut microbiota and mucosal immunity [21]. Furthermore, glucose is also a preferred source of carbon for microbial metabolism. The gut microbiota is enriched in glucose-metabolism-related pathways [24]. Glucose as a carbohydrate supplement is a good choice when animals have large energy requirements.

As the main components of bile, bile acid (BA) maintains stable metabolism and circulation under normal physiological conditions. BA is synthesized from cholesterol and stored in the gallbladder [25]. The gallbladder expels BAs into the small intestine to promote the digestion and absorption of dietary fat and cholesterol [26]. Approximately 95% of BAs in the lumen, which are retaken at the distal ileum by the apical Na^+^-dependent bile salt transporter (ASBT) [27,28], are transported into the blood circulation and then into the liver cells [29]. Complete enterohepatic circulation helps to maintain bile acid homeostasis. In fact, BAs also interact positively with gut microbiota [30]. The gut microbiota is involved in BA metabolism, which is indispensable for the biotransformation of primary BAs into secondary BAs [31]. It has been reported that BAs are extremely important effectors under conditions of sustained brown fat activation, and this metabolic program contributes to thermogenesis during cold exposure [32]. Therefore, we have to focus on changes in the BA circulation and gut microbiota during prolonged cold exposure.

Based on the above research background, this study proposes the following hypotheses: Under chronic cold stress conditions, dietary supplementation with full-fat rice bran or glucose can improve the growth and health of cold-exposed pigs through the following pathways: Full-fat rice bran enhances the body’s antioxidant capacity by virtue of its rich antioxidant-active substances (such as tocopherol and ferulic acid), and at the same time regulates the gut microbiota structure and promotes the production of SCFAs through dietary fiber. Glucose reduces cold stress-induced lipolysis and metabolic disorders by supplementing energy substrates, thereby improving intestinal barrier function and bile acid circulation; furthermore, both diets can enhance the cold adaptation ability of pigs by regulating the gut microbiota–bile acid interaction network, and ultimately achieve the improvement or maintenance of growth performance.

In this research, we aimed to assess the regulatory effects of dietary full-fat rice bran or glucose on growth performance, antioxidant capacity, bile acid circulation, and gut microbiota in cold-exposed pigs, providing a new basis for solving the adverse impacts of long-term cold on pigs by means of nutritional regulation.

## 2. Methods

### 2.1. Experimental Design, Animals, and Diets

**Figure 1:**
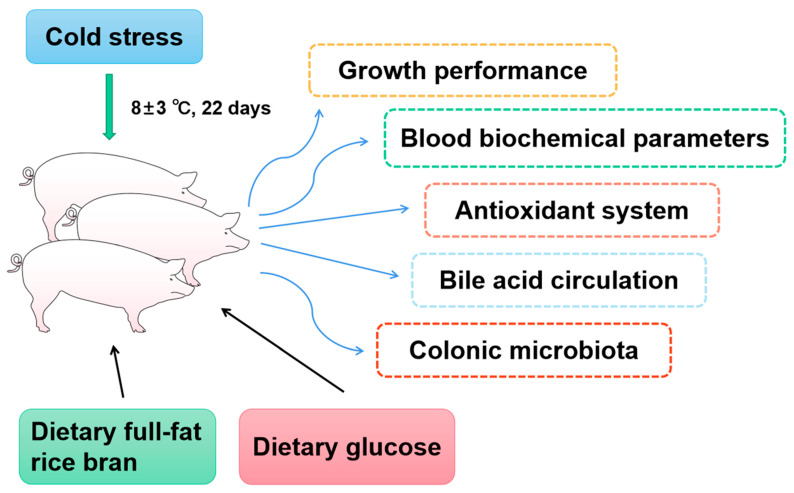
**The technical route of this trial.** Effects of dietary full-fat rice bran or glucose on growth performance, blood biochemical parameters, blood antioxidant, bile acid circulation, and colonic microbiota of pigs during cold stress.

Eighteen healthy Yorkshire sows (body weight: 23.74 ± 0.14 kg) were selected from a commercial farm and divided into three groups based on their body weight. All Yorkshire pigs were individually housed in stainless-steel metabolic cages (1.78 m × 0.84 m × 1.40 m). All Yorkshire pigs were housed individually in stainless-steel metabolic cages and experienced natural ambient temperatures (8 ± 3 °C) of the pig houses during the winter (Figure 1). This study comprised 3 dietary treatments: the basal diet (low-temperature control group, C-LT), basal diet supplemented with full-fat rice bran (B-LT group), and glucose diet (G-LT group). All diets used in the experiment were in powdered form. Three diets in this research were formulated to meet the NRC requirements (NRC, 2012) [33]. The ingredient and nutritional levels are listed in Table 1. All diet foods were kept in a cool and dry storeroom. All pigs were allowed access to water and feed ad libitum during the entire research period (22 days). The pig houses and stainless-steel metabolic cages were cleaned and sanitized before the experiment.

### 2.2. Growth Performance

We recorded the body weight and food intake of pigs to calculate average daily gain (ADG), average daily food intake (ADFI), and feed/gain (*F/G*). The growth performance was characterized according to the previous calculation formulae [34].

### 2.3. Sample Collections

On the morning of day 23 of the experiment, venous blood was collected from all experimental pigs and transferred into heparinized anticoagulant tubes. Subsequently, the blood was centrifuged at 3500 revolutions per minute (rpm) for 10 min at 4 °C. After centrifugation, the separated serum was aliquoted and stored at −20 °C for subsequent detection and analysis. After fasting for 12 h, pigs were electrocuted and slaughtered. About 2 g of liver (median lobe) was collected in cryo-storage tubes, and the mucosa samples of duodenum (middle section), jejunum (middle section), ileum (middle section), and colon (middle section) were scraped by glass microscope slides. Obtained samples were stored in a −80 °C refrigerator.

### 2.4. Biochemical Analysis, Antioxidant Enzyme Activity

Plasma total cholesterol (CHOL), triglycerides (TG), alanine aminotransferase (ALT), aspartate amino-transferase (AST), AST/ALT, high-density lipoprotein-cholesterol (HDL-C), low-density lipoprotein-cholesterol (LDL-C), total protein (TP), albumin (ALB), globulin (GLB), ALB/GLB (*A*/*G*), total bilirubin (TBIL), and total bile acid (TBA) were characterized by an automated biochemical analyzer (Roche, Cobus-Mira-Plus, Roche Diagnostic System Inc., Basel, Switzerland), according to the manufacturer’s guidelines. These indicators were examined by the commercial kits (Nanjing Jiancheng Bioengineering Institute, Nanjing, China). The methane dicarboxylic aldehyde (MDA), superoxide dismutase (SOD), glutathione peroxidase (GSH-px), and total antioxidant capacity (T-AOC) in plasma were determined via commercial kits from the Nanjing Jiancheng Bioengineering Institute, according to the manufacturer instructions.

### 2.5. Quantitative RT-PCR Analysis

Total RNA of the liver, ileum mucosa, and colonic mucosa was extracted by Trizol Reagent (Takara, Beijing, China). The total RNA from the samples was reverse-transcribed into cDNA by a PrimeScript ™ RT reagent kit with a gDNA Eraser Kit (Takara, Beijing, China). The RT-qPCR was carried out by SYBR mix (Takara Bio Inc., Kyoto-shi, Japan) to measure mRNA expression. The β-actin was served as a reference gene. And the relative mRNA expression was normalized to the control gene (β-actin) and determined using the 2^–ΔΔCt^, according to the previous method [35]. Information on all the primers in this trial is summarized in Table 2.

### 2.6. 16S rDNA Gene Sequencing Analysis

A QIAamp DNA Stool Mini kit (Qiagen, Hilden, Germany) was used to extract the total bacterial DNA from colonic contents (*n* = 6). The V4 hypervariable region of the 16S rDNA gene was PCR-amplified using the primers 515F and 806R, according to the previous method [36]. Next, paired-end sequencing was carried out on an Illumina HiSeq 2500 platform (Bioacme Coa, Wuhan, China). Raw reads in this study were filtered and merged as raw tags through the FASTP. Raw tags were filtered to produce clean tags. After that, clean tags were used for clustering to get operational taxonomic units after quality filtering. Then, the abundance of operational taxonomic units was conducted.

### 2.7. Quantification of Short-Chain Fatty Acids (SCFAs) in Colonic Contents

An amount of 20 mg of colonic contents of each pig was placed in the EP tube including 1 mL phosphoric acid solution (0.5% *v*/*v*). Then, colonic contents were mixed and extracted for 10 min. Next, they were sonicated for 5 min and then centrifuged (10 min, 6000× *g*) at 4 °C [34]. And 0.1 mL supernatant of each sample was taken and transferred to a centrifugal tube. Then, 0.5 mL MTBE solution, which contained an internal standard, was added to these centrifugal tubes. Afterward, samples were vortexed for 3 min and sonicated for 5 min. Then, they were centrifuged for 10 min at 12,000× *g* at 4 °C. After centrifugation, about 0.3 mL of supernatant was filter-sterilized with a 0.22 μm filter for gas chromatography–mass spectrometry analysis.

### 2.8. Statistical Analysis

Each pig was considered to be a statistical unit. Data were evaluated for the normality and homogeneity of variances. Then, data were analyzed by a one-way ANOVA, followed by Tukey’s multiple comparisons through SPSS 22.0. All data in this study were visualized by GraphPad Prism (version 8.0, GraphPad, San Diego, CA, USA). Data involved in this research were displayed as the means ± standard error of measurement (SEM): “*” means *p* < 0.05; “**” means *p* < 0.01.

## 3. Results

### 3.1. Effects of Full-Fat Rice Bran or Glucose on Growth Performance of Cold-Exposed Pigs

**Figure 2:**
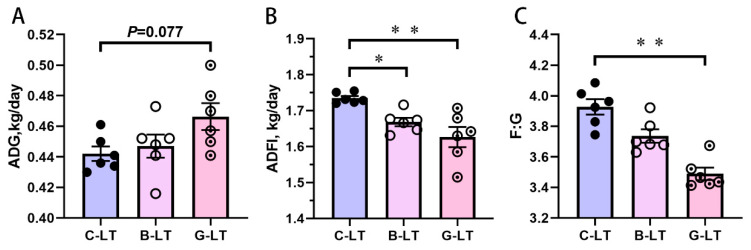
**Effects of dietary Full-fat rice bran or glucose on growth performance of pigs during cold stress.** (**A**) Average daily gain (ADG). (**B**) Average daily feed intake (ADFI). (**C**) Feed/gain (F/G). Data are displayed as the means ± standard error of measurement (SEM): “*” means *p* < 0.05; “**” means *p* < 0.01. C-LT (low-temperature control group), B-LT (basal diet supplemented with full-fat rice bran group), G-LT (basal diet supplemented with glucose group).

Compared with that in the C-LT group, ADG in the G-LT group displayed a tendency to increase (*p* = 0.077, Figure 2A). There was no significant change in ADG in the B-LT group (*p* > 0.05, Figure 2A). Dietary full-fat rice bran and glucose induced a decrease in ADFI during cold exposure (*p* < 0.05, Figure 2B). The F/G in the G-LT group was lower than that in the C-LT group (*p* < 0.01, Figure 2C), while the F/G in the B-LT group was not affected (*p* > 0.05, Figure 2C).

### 3.2. Effects of Full-Fat Rice Bran or Glucose on Plasma Biochemical Parameters of Cold-Exposed Pigs

As shown in Table 3, the biochemical parameters in the plasma of pigs were detected. Compared with those in the C-LT group and the B-LT group, the plasma ALT levels in the G-LT group were decreased significantly (*p* < 0.05). The plasma TG concentration was increased in the B-LT group, compared to that in the C-LT group (*p* < 0.05). Compared with that in the C-LT group, the TBA concentration in the G-LT group tended to be increased (*p* < 0.05), but the TBA concentration in the B-LT group did not change (*p* > 0.05). Neither treatment significantly affected other blood biochemical parameters of cold-exposed pigs.

### 3.3. Effects of Full-Fat Rice Bran or Glucose on Plasma Antioxidant Parameters of Cold-Exposed Pigs

**Figure 3:**
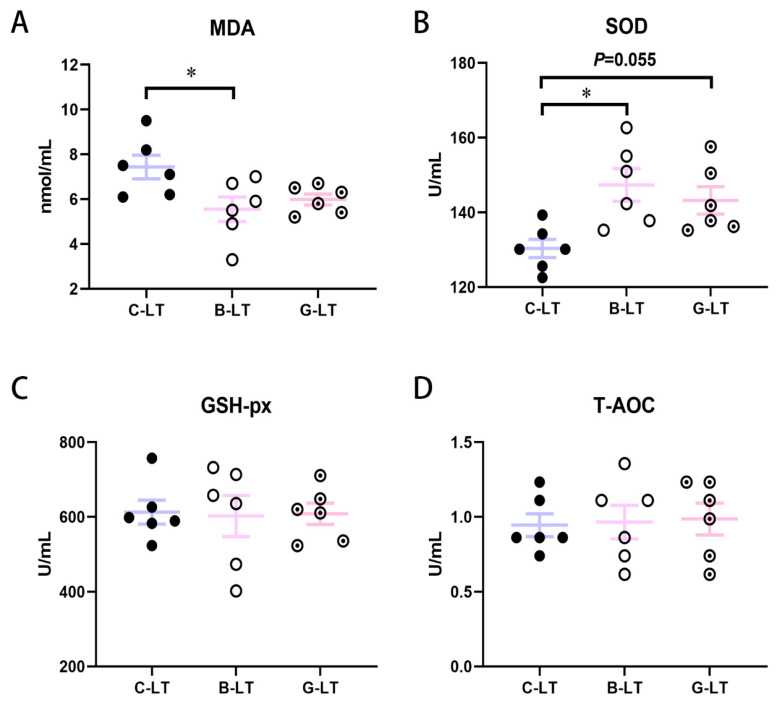
**Effects of dietary full-fat rice bran or glucose on blood biochemical parameters of pigs during cold stress.** (**A**) Methane dicarboxylic aldehyde (MDA). (**B**) Superoxide dismutase (SOD). (**C**) Glutathione peroxidase (GSH-px). (**D**) Total antioxidant capacity (T-AOC). Data are displayed as the means ± standard error of measurement (SEM): “*” means *p* < 0.05.

Figure 3 showed plasma antioxidant parameters in Yorkshire pigs of the three groups. The MDA concentration in the B-LT group was significantly lower than the in the C-LT group (*p* < 0.05, Figure 3A). However, the SOD activity in the B-LT group was higher than that in the C-LT group (*p* < 0.05, Figure 3B). The SOD activity of the G-LT group showed an increasing trend compared to that in the C-LT group (*p* = 0.055, Figure 3B). The GSH-px and the T-AOC in pigs with cold exposure were not regulated by dietary full-fat rice bran and glucose (*p* > 0.05, Figure 3C,D).

### 3.4. Effects of Dietary Full-Fat Rice Bran or Glucose on Bile Acid (BA) Transport in the Ileal Mucosa of Cold-Exposed Pigs

**Figure 4:**
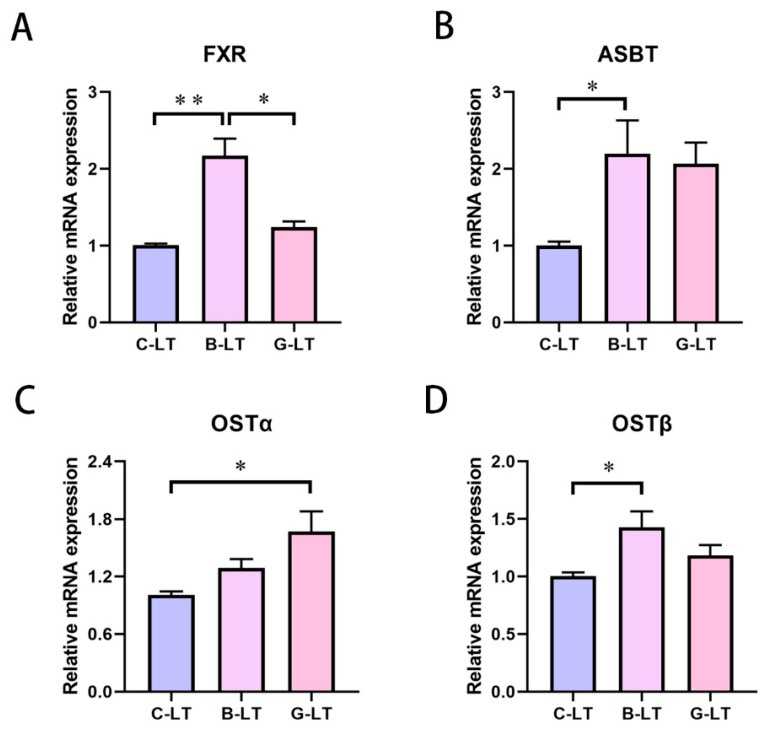
**Effects of dietary full-fat rice bran or glucose on ileal-bile-acid-related factors of pigs during cold stress.** (**A**) Farnesoid X receptor (FXR). (**B**) Apical sodium-coupled bile acid transporter (ASBT). (**C**) Organic solute transporter α (OSTα). (**D**) Organic solute transporter β (OSTβ). Data are displayed as the means ± standard error of measurement (SEM): “*” means *p* < 0.05; “**” means *p* < 0.01.

Due to changes in plasma TBA concentrations, we focused on bile acid (BA) circulation under the two dietary treatments during cold exposure. The ileum is the main site of BA reabsorption. Therefore, we represented the expression of BA receptor and genes involved in the BA transport and excretion of the ileal mucosa. Compared with that in the C-LT group and the G-LT group, the Farnesoid X receptor (FXR) mRNA expression in the ileal mucosa of the B-LT group was upregulated (*p* < 0.05, Figure 4A). The apical sodium-coupled bile acid transporter (ASBT) and organic solute transporter β (OSTβ) mRNA expression was enhanced in the ileal mucosa of the B-LT group compared with that in C-LT group (*p* < 0.05, Figure 4B,D). In addition, the mRNA expression level of organic solute transporter α (OSTα) in the ileal mucosa of the G-LT group was higher than that in the C-LT group (*p* < 0.05, Figure 4C).

### 3.5. Effects of Dietary Rice Bran or Glucose on Hepatic Bile Acid Synthesis in Cold Exposed Pigs

**Figure 5:**
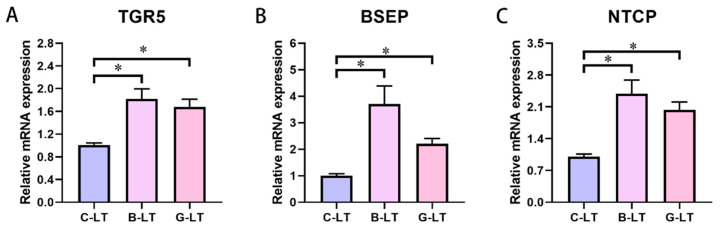
**Effects of dietary full-fat rice bran or glucose on liver-bile-acid-related factors of pigs during cold stress.** (**A**) G-protein-coupled bile acid receptor (TGR5). (**B**) Bile salt excretory pump (BSEP). (**C**) Na^+^ taurocholate cotransporter polypeptide (NTCP): “*” means *p* < 0.05.

Subsequently, we detected the expression of bile-acid-related genes and proteins in the liver of pigs (Figure 5). The G-protein-coupled bile acid receptor (TGR5), Na^+^ taurocholate cotransporter polypeptide (NTCP), and bile salt excretory pump (BSEP) mRNA expression in the liver of the B-LT group and G-LT group was enhanced compared with that in the C-LT group (*p* < 0.05, Figure 5A–C).

### 3.6. Effects of Dietary Full-Fat Rice Bran or Glucose on Colonic Microbiota in Cold-Exposed Pigs

**Figure 6:**
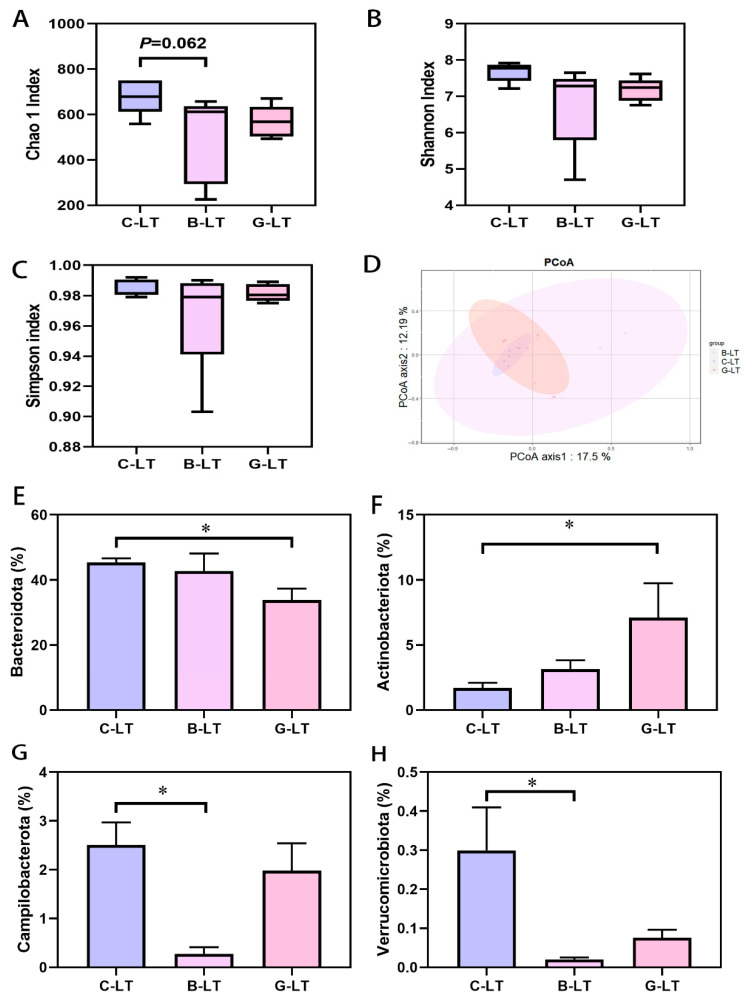
Effects of dietary full-fat rice bran or glucose on the diversity and phylum level of colonic microbiota in pigs during cold stress (**A**) Chao 1 index. (**B**) Shannon index. (**C**) Simpson index. (**D**) PCoA analysis. (**E**) *Bacteroidetes*. (**F**) *Acfinobacteria*. (**G**) *Campilobacterota*. (**H**) *Verrucomicrobiota*. Data are displayed as the means ± standard error of measurement (SEM): “*” means *p* < 0.05.

Next, we focused on changes in the gut microbiota of cold-exposed pigs regulated by these two diets. We first observed the alpha diversity index of the colonic microbiota (Figure 6). Compared with that in the C-LT group, the Chao1 index of colonic microbiota in the B-LT group showed a decreasing trend (0.05 < *p* < 0.1, Figure 6A), and no changes were detected in the Shannon and Simpson indices of the B-LT group (*p* > 0.05, Figure 6B,C). The alpha diversity of colonic microbiota in cold-exposed pigs was not altered by dietary glucose. PCoA analysis showed that dietary full-fat rice bran and glucose significantly changed the microbiota (Figure 6D). Compared to that in the C-LT group, the abundance of *Bacteroidetes* in the colon microbiota of the G-LT group was decreased, while the relative abundance of *Actinobacteria* was significantly increased (*p* < 0.05, Figure 6E,F). In addition, the lower abundance of *Campilobacterota* and *Verrucomicrobiota* was detected in the colon microbiota of the B-LT group compared to that in the C-LT group (*p* < 0.05, Figure 6G,H).

**Figure 7:**
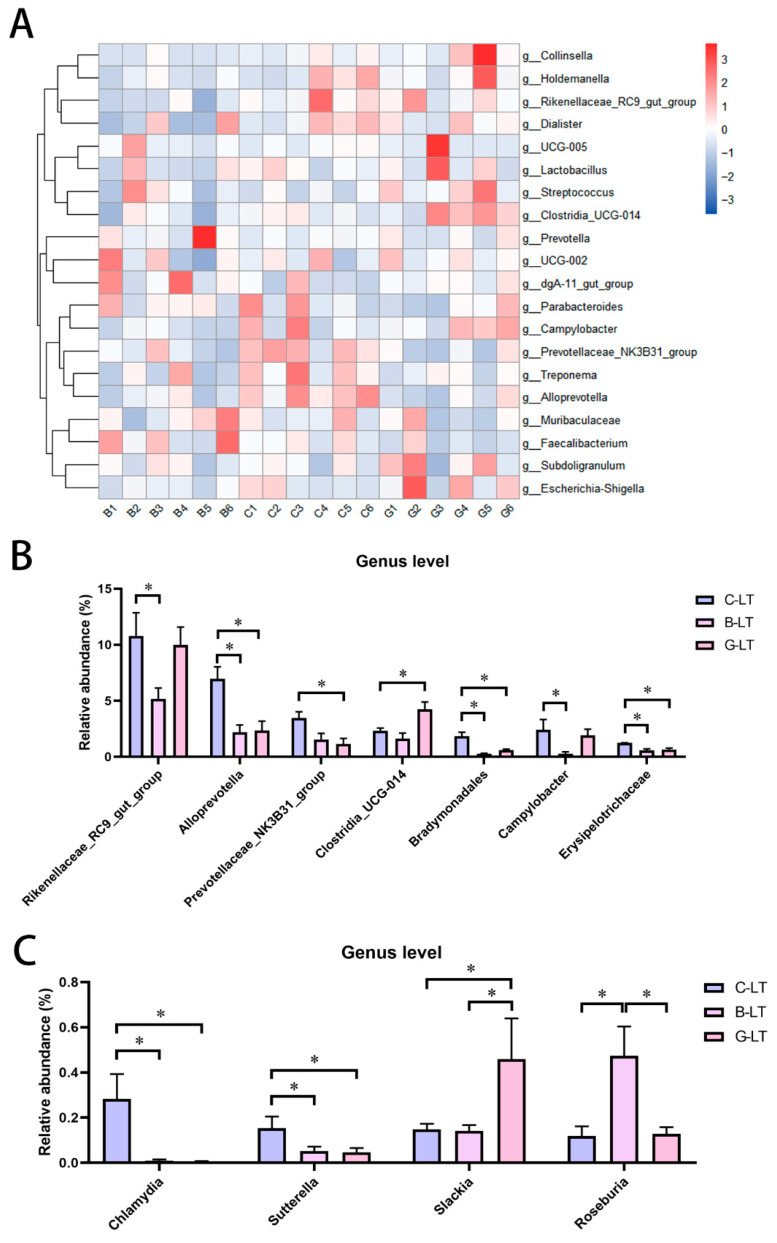
Effects of dietary full-fat rice bran or glucose on general-level abundance of colon microbiota of pigs during cold stress. (**A**) Heatmap of the relative abundance of colonic microbiota at the top 25 genus level. (**B**) Relative abundance of colonic microbiota at the level of the top 25 genera with significant changes. (**C**) Changes in the relative abundance of bacteria genera with significant changes in colonic microbiota beyond the level of the top 25 genera. Data are displayed as the means ± standard error of measurement (SEM): “*” means *p* < 0.05.

The relative abundance of colonic microbiota at the top 25 genus level was further analyzed (Figure 7A). The relative abundance of *Rikenellaceae_RC9_gut_group* and *Campylobacter* in the B-LT group was lower than that in the C-LT group (*p* < 0.05, Figure 7B). Moreover, the relative abundance of *Alloprevotella*, *Bradymonadales*, and *Erysipelotrichaceae* in the colon microbiota was reduced in both the B-LT group and the G-LT group, compared with that in the C-LT group (*p* < 0.05, Figure 7B). In addition, the abundance of *Prevotellaceae_NK3B31_group* in the G-LT group was lower than that in the C-LT group, while the abundance of *Clostridia_UCG-014* was higher than that in the C-LT group (*p* < 0.05, Figure 7B). In addition to the relative abundances of the top 25 genera, the relative abundance of *Chlamydia* and *Sutterella* was reduced in the B-LT group and the G-LT group compared to that in the C-LT group (*p* < 0.05, Figure 7C). *Slackia* was highly enriched in the G-LT group, and *Roseburia* was highly enriched in the B-LT group (*p* < 0.05, Figure 7B). Other non-prominent bacterial genera are detailed in Supplementary Material Figure S1.

### 3.7. Effects of Dietary Full-Fat Rice Bran or Glucose on Colonic SCFAs in Cold-Exposed Pigs

**Figure 8:**
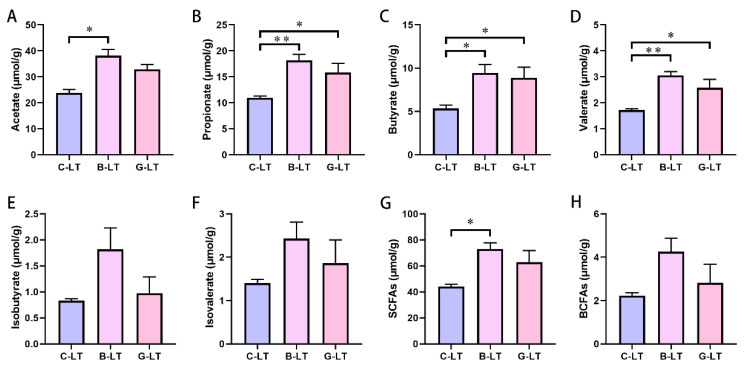
**Effects of dietary full-fat rice bran or glucose on short-chain fatty acids (SCFAs) of pigs during cold stress.** (**A**) Acetate. (**B**) Propionate. (**C**) Butyrate. (**D**) Valerate. (**E**) Isobutyrate. (**F**) Isovalerate. (**G**) Total SCFAs. (**H**) Total branched-chain fatty acids (BCFAs). Data are displayed as the means ± standard error of measurement (SEM): “*” means *p* < 0.05; “**” means *p* < 0.01.

We reveal the changes in the concentration of SCFAs in the colonic content of cold-exposed pigs. Our data showed that the concentration of acetate, propionate, butyrate, valerate, and SCFAs in the B-LT group were increased compared to that in the C-LT group significantly (*p* < 0.05, Figure 8A–D,G). Moreover, propionate, butyrate, and valerate concentrations were also increased in the G-LT group as compared to those in the CON group (*p* < 0.05, Figure 8B–D). However, there was no significant difference in the concentrations of isobutyrate, isovalerate, and total branched-chain fatty acids (BCFAs) (*p* > 0.05, Figure 8E,F,H).

## 4. Discussion

Chronic cold stress has always been one of the great threats to animals in northern alpine areas. Growing pigs are extremely sensitive to temperature changes. There is no doubt that prolonged cold exposure increases energy expenditure of growing pigs. Prolonged low temperatures retard growth performance of pigs and increase the feed/gain ratio to reduce the economic benefits of farms [37]. Even chronic cold stress increases the mortality of pigs [38]. Therefore, adjusting the dietary ingredient composition could serve as an effective strategy for mitigating the adverse effects of raising pigs during cold exposure. Rice bran is a kind of feed ingredient rich in protein, lipids, dietary fiber, and vitamins [10], and glucose is the most common quick energy supply [39]. In this study, 18 healthy Yorkshire sows were selected. Using pigs of a single gender helps reflect the objectivity of the experimental results; furthermore, females have a more stable metabolic baseline, which better ensures the objectivity of the overall experimental results. Here, we sought to evaluate the regulatory effects of two dietary strategies (dietary full-fat rice bran and dietary glucose) on growth, antioxidant, bile acid circulation, and colonic microbiota in cold-exposed Yorkshire pigs.

Dating back to the last century, early evidence has suggested that chronic cold stress induces active feeding behavior in pigs, accompanied by significant increases in feed intake [40]. Even so, however, the growth rate of pigs is inhibited [41]. Therefore, the improvement in growth performance of cold-exposed pigs is the primary indicator of general concern. In the current study, we found beneficial impacts of dietary full-fat rice bran and dietary glucose on growth performance of cold-exposed pigs. Dietary glucose supplementation, similar to previous results of dietary supplementation with fats and other energy supplements [20,42], can significantly increase the ADG of growing pigs under cold exposure, while significantly reducing ADFI and F/G. As a carbohydrate supplement, glucose can improve the efficiency of pigs’ utilization of feed energy, thereby promoting the growth rate of pigs during cold exposure. In addition, dietary rice bran supplementation can also reduce the ADFI of growing pigs under cold exposure; however, in terms of the effect on improving pigs’ growth performance alone, its effectiveness is inferior to that of glucose supplementation. The main reason for this difference is most likely the variation in energy values between the two diets.

The evidence to date suggests that chronic cold stress mainly causes oxidative stress damage in animals, especially the lipid peroxidation [43,44]. During cold exposure, the animal’s antioxidant system weakens, and the animal gradually loses the ability to adapt to the cold. Reactive oxygen species (ROS) excessive accumulation causes lipid peroxidation. MDA is widely regarded as one of the lipid peroxidation parameters and a marker of oxidative-stress-related damage [45,46]. Antioxidant enzymes such as SOD and GSH-px regulate ROS homeostasis [47]. SODs are the primary defense against ROS in animals. Superoxide radicals are converted to molecular oxygen and H_2_O_2_ by SODs [48]. Previous studies imply that chronic cold stress increases MDA levels and inhibits SOD activity [44]. Studies have shown that dietary supplementation with full-fat rice bran can alleviate the significant increase in liver MDA levels caused by cold exposure, which is consistent with the results of our study. The results of this study indicate that dietary supplementation with full-fat rice bran can significantly reduce the plasma MDA concentration and significantly increase the plasma SOD activity in cold-exposed pigs. These results implied that dietary full-fat rice bran reduced the risk of chronic cold-stress-induced oxidative stress in pigs. Many bioactive molecules with antioxidant activity, such as panquinone-10, α-tocopherol, γ-glutamate, and phytosterol, are enriched in rice bran, which help to improve the antioxidant level of animals [49]. These antioxidants in rice bran are likely to play a necessary role in improving SOD activity in cold-exposed pigs. In addition, although the glucose diet did not inhibit plasma MDA levels in cold-exposed pigs, it increased SOD activity to some extent, which also helped growing pigs adapt to prolonged cold exposure.

Bile acids (BAs) are found mainly in the bile of animals. The major function of BAs is to participate in the emulsification, absorption, and digestion of lipids [50]. Bile acids are produced in the liver and excreted into the intestine. Moreover, the gut microbiota converts primary BAs into secondary BAs via 7α-dehydroxylation and deconjugation [51]. BAs in the intestinal lumen are absorbed in the terminal ileum and transferred to the liver through enterohepatic circulation. The homeostasis of bile acid circulation is essential for energy metabolism and immunity. This homeostasis is coordinated by the synthesis, reabsorption, and excretion of BAs [52]. FXR is highly expressed in the intestine and liver and is widely recognized as a conductor of BA balance [53]. Activated by excess bile acids in the gut, FXR promotes the transcription of fibroblast growth factor 15/19, and then activates FGF receptor 4 in the liver to inhibit CYP7A1 expression, thereby limiting bile acid synthesis [54]. This is a classic FXR-mediated BA negative-feedback regulation mechanism. Meanwhile, FXR enhances the expression of OSTα and OSTβ related to bile acid excretion [55,56]. Authoritative evidence suggests that bile-acid-mediated metabolic processes help animals adapt to low temperatures [32]. In our study, we observed an increase in plasma TBA concentration in the G-LT group, which led us to pay close attention to changes in bile acid circulation. Interestingly, further analysis of genes regulating bile acid homeostasis in the ileum and liver revealed that in cold-exposed pigs, both full-fat rice bran and glucose supplementation activated the hepatic FXR pathway—with a concurrent decrease in CYP7A1 expression. Notably, however, high FXR mRNA levels were only detected in the ileal mucosa of the B-LT group. Moreover, with the increased expression of OSTβ in the B-LT group and OSTα in the G-LT group, the intestinal bile acid excretion of cold-exposed pigs was enhanced. More surprisingly, the relative mRNA expression of ASBT was ameliorated in the B-LT group, compared to that in the C-LT group. ASBT, as an integral brush border membrane glycoprotein enriched in the ileum, regulates BA reabsorption [57]. Although some evidence indicates that ASBT is inhibited by FXR-mediated negative-feedback regulation of BAs [58], the increased expression level of ASBT mRNA in the B-LT group might promote BA reabsorption. The uptake of bile acids is regulated by NTCP, and the BSEP delivers the BAs to the bile ducts in the liver [59,60]. Both the rice bran diet and the glucose diet enhanced the expression of these two genes in the liver of cold-exposed pigs, indicating active BA circulation fluxes. Studies have shown that bile acids regulate lipid metabolism by activating TGR5 [61]. Both the rice bran diet and the glucose diet provided more energy substrates, including glucose and triglycerides. This may help the pigs adapt to the cold. Interestingly, we found that the expression of FXR in the ileum of the rice bran group was significantly higher than that in the other two groups. However, there were certain differences between the expression of TGR5 and FXR in the liver: the expression levels of both in the two treatment groups were significantly higher than those in the control group, but there was no significant difference between the two treatment groups. This may be attributed to the fact that rice bran contains components such as dietary fiber and unsaturated fatty acids, which are not easily digested and absorbed by the small intestine. These components can reach the ileum directly and be fermented by intestinal microbiota, thereby altering the microbiota structure, indirectly affecting bile acid transformation, and consequently influencing FXR expression. In contrast, TGR5 is not only activated by bile acids but also closely related to energy metabolism (such as thermogenesis and lipid breakdown). Under cold stress, both treatment groups met the pigs’ thermogenic needs by supplementing energy substrates. The activating effect of this “metabolic signal” on hepatic TGR5 masked the potential impact of differences in dietary components, resulting in no significant difference in hepatic TGR5 between the two treatment groups. However, due to the lack of a normal temperature control group, our experimental results apply to the finding that supplementing amino acids and full-fat rice bran in the diet under cold conditions can enhance bile acid circulation and help animals resist cold.

Secondary bile acids are produced by the processing of intestinal microbiota. They can improve the health of animal organisms and alleviate cold stress [32,62,63]. The increase in Actinobacteria in the glucose group may be related to the production of secondary bile acids [64]. Studies have shown that an increase in the abundance of *Erysipelotrichaceae* in the ileum is accompanied by a significant decrease in the level of secondary bile acids, suggesting that *Erysipelotrichaceae* may be involved in the regulation of secondary bile acid levels [65].

The gut microbiota is closely related to the immunity and metabolism of animals. We focused on the regulatory effects of full-fat rice bran and glucose on the colonic microbiota in cold-exposed pigs. Generally, the Chao 1 index and ACE index indicate microbial diversity [66]. Dietary full-fat rice bran tended to decrease the Chao1 and ACE indices of colon microbiota in pigs, suggesting that adding full-fat rice bran during cold stress might reduce microbiota diversity. We also observed that dietary rice bran and glucose altered the phylum level abundance of the colon microbiota. Compared to that in the C-LT group, *Bacteroidetes* abundance in the colon microbiota of the G-LT group was decreased, while *Actinobacteria* was increased. When the ratio of *Firmicutes* to *Bacteroidetes* increases, fat deposition is increased, which means better use of dietary energy [67,68]. The *Actinobacteria* contains some well-known probiotics, such as *Bifidobacterium* [69]. Apparently, dietary glucose promoted energy deposition in cold-exposed pigs and may have increased probiotic abundance. In addition, in our research, a lower abundance of *Campilobacterota* was observed in the colon microbiota of the B-LT group compared to that in the C-LT group, which was a major contributor to ulcerative colitis [70]. Further analysis at the genus level implied that the *Rikenellaceae_RC9_gut_group* and *Campylobacter* abundance in the B-LT group was lower than that in the C-LT group among the top 25 genera. *Rikenellaceae_RC9_gut_group* is thought to increase the sensitivity of the gut to inflammation [71]. Additionally, it showed correlations with levels of taurolithocholic acid (TLCA), taurodeoxycholic acid (TDCA), and taurocholic acid (TCA) in the bloodstream [72]. *Campylobacter* is a common enteropathogenic bacterium that induces intestinal dysfunction in animals [73]. Moreover, *Alloprevotella* and *Erysipelotrichaceae* abundance in the colon microbiota was reduced in both the B-LT group and the G-LT group. Actually, *Alloprevotella* is a probiotic that produces SCFAs [74]. However, we found that both dietary rice bran and glucose contributed to the content of SCFAs in colon contents during cold conditions. *Alloprevotella* may not be a major contributor to SCFAs during either dietary intervention. Some bacteria in *Erysipelotrichaceae*, such as *Carbapenem-resistant Enterobacteriaceae*, have been proven able to promote intestinal inflammation [75]. Thus, the rice bran diet and glucose supplement diet can inhibit some pathogens. In addition, in this research, the abundance of *Prevotellaceae_NK3B31_group* in the G-LT group was less than that in the C-LT group. *Prevotellaceae_NK3B31_group* was negatively correlated with TNF-α expression and salmonella infection [76,77]. Thus, glucose supplementation mitigated the risk of *Prevotellaceae_NK3B31_group* during chronic cold stress. We also found that *Chlamydia* and *Sutterella* abundance was reduced in the B-LT group and the G-LT group. *Roseburia* was highly enriched in the B-LT group. *Chlamydia* and *Sutterella* are pathogens of great concern in the medical field [78,79]. *Roseburia* has been shown to produce butyrate to protect the gut [80], thereby facilitating cold adaptation. Remarkably, there has been shown an enrichment of *Rikenellaceae_RC9_gut_group*, *Alloprevotella*, *Prevotellaceae_NK3B31_group*, and *Erysipelotrichaceae* within the gut of cold-exposed piglets [72]. In the B-LT group, there was a reduction presence in the abundance of *Erysipelotrichaceae, Prevotellaceae_NK3B31_group*, *Alloprevotella*, and *Rikenellaceae_RC9_gut_group*. Similarly, the G-LT group exhibited a reduced abundance of *Erysipelotrichaceae*, *Prevotellaceae_NK3B31_group*, and *Alloprevotella*. These findings collectively indicate that diets rich in full-fat rice bran and glucose might exert a partial reversal effect on the cold-stress-induced alterations in the colon microbiota. Interestingly, we have found results that corroborate and are similar to those of this experiment in other animal species: for instance, an abundance of *Roseburia* decreases in cold-exposed rats [81], and an abundance of *Bacteroidetes* in cold-exposed mice [82]. Gut microbiota ferment carbohydrates into SCFAs, which play a critical role against pathogenic infection in the intestinal tract [83]. Acetate, propionate, butyrate, and valerate account for more than 90% of the total SCFAs. We found that dietary rice bran increased the concentrations of acetate, propionate, butyrate, and valerate in colon contents of pigs. Moreover, dietary glucose increased the propionic acid, butyric acid, and valerate levels in colon contents. It follows that both diets improved the intestinal SCFAs of pigs during chronic cold exposure. Overall, dietary full-fat rice bran and glucose modulated the colonic microbiota and SCFAs of cold-exposed pigs.

## 5. Conclusions

The present study demonstrated that dietary full-fat rice bran or glucose supplementation improves colonic microbiota and short-chain fatty acids in cold-exposed pigs. Compared with the two dietary strategies, dietary glucose is more beneficial to the growth performance of cold-exposed pigs. Dietary whole-fat rice bran is more beneficial to the antioxidant system. In addition, dietary glucose significantly increased the plasma total bile acid concentration in cold-exposed pigs, and enhanced bile acid excretion by activating the hepatic Farnesoid X receptor pathway and upregulating organic solute transporter alpha expression. In contrast, although dietary full-fat rice bran did not alter the total bile acid level, it regulated the bile acid reabsorption process by upregulating the expression of Farnesoid X receptor and apical sodium-dependent bile acid transporter in the ileum. Notably, both diets participated in the regulation of bile acid homeostasis through different pathways, thereby enhancing the pigs’ adaptability to the cold environment. Our findings highlight the potential use of full-fat rice bran and glucose to regulate the growth and health of cold-exposed pigs.

## Figures and Tables

**Table 1:** Composition of experimental diets.

Basic Diet Ingredients	C-LT (%)	B-LT (%)	G-LT (%)
Corn	73.00	56.97	60.68
Soybean meal, de-hulled	15.30	11.58	17.53
Full-fat soybean meal, puffed	5.00	5.00	5.00
Full-fat rice bran		20.00	
Fish meal	2.00	2.00	2.00
Soybean oil	1.00	1.00	1.00
Glucose			10.00
L-Lysine	0.39	0.38	0.35
DL-Methionine	0.04	0.04	0.05
L-Threonine	0.12	0.11	0.11
L-Tryptophan	0.02	0.02	0.01
Calcium hydrogen phosphate	1.19	0.45	1.25
Limestone	0.66	1.05	0.62
Salt	0.28	0.40	0.40
Premix ^A^	1.00	1.0	1.00
**Nutrient levels ^B^**			
NE ^C^, Mcal/kg	2.50	2.52	2.63
Crude protein	16.03	16.03	16.03
Lysine	0.98	0.98	0.98
Methionine	0.29	0.29	0.29
Threonine	0.60	0.60	0.60
Leucine	0.17	0.17	0.17
Calcium	0.66	0.66	0.66
Total phosphorus	0.56	0.70	0.56
Available phosphorus	0.33	0.27	0.34
Sodium	0.14	0.20	0.19
Chlorine	0.19	0.27	0.26

Premix ^A^, 160 mg; Cu, 150 mg; Mn, 40 mg; Zn, 140 mg; Se, 0.4 mg; I, 0.5 mg; vitamin A, 8000 IU; vitamin D3, 2000 IU; vitamin E, 30 mg; vitamin B1, 1.60 mg; vitamin B2, 5.00 mg; vitamin B6, 5.00 mg; vitamin B12, 0.01 mg; pantothenic acid, 20 mg; niacin, 15 mg; biotin, 0.05 mg. ^B^ Nutrient levels were calculated values. ^C^ NE: net energy.

**Table 2:** The real-time PCR primers.

Gene	GenBank ID	Primer Sequences (5′ to 3′)
β-actin	AY550069	F: ATGCTTCTAGGCGGACTGT
		R: CCATCCAACCGACTGCT
FXR	NM_001287412.1	F: TATGAACTCAGGCGAATGCCTGCT
		R: ATCCAGATGCTCTGTCTCCGCAAA
ASBT	NM_001244463.1	F: TTGGCCTACTGGGTTGATGG
		R: AGATTAAGAGGCACAGCGGC
OSTα	NM_001244266.1	F: TGTACAAGAACACTCGCTGC
		R: GAACACACACACTATCGTGGG
OSTβ	XM_003121716.5	F: ACTGAGGTCCTCTCCAGTCC
		R: CGGCTGTCACCTCTTGAATG
TGR5	XM_013984487.1	F: TGCTGTCCCTCATCTCATTGG
		R: TGTGTAGCGATGATCACCCAG
BSEP	U20587.1	F: CGGGCCATCGTACGAGAT
		R: CCGTCTTTTCGCTTTCTGTGT
NTCP	AK232743	F: GCCACCTCCTCCCTTATGC
		R: GGCGGAAAAGAGCAGAAAGA

**Table 3:** Effects of full-fat rice bran and glucose on blood biochemical parameters of cold-exposed pigs.

Items	C-LT	B-LT	G-LT	SEM ^d^	*p*-Value
TP (g/L)	69.38	66.72	67.65	1.49	0.782
ALB (g/L)	33.88	33.53	32.92	0.56	0.796
GLB (g/L)	35.5	33.16	34.73	1.72	0.867
ALT (IU/L)	39.25 ^a^	38.88 ^ab^	28.05 ^c^	1.91	0.014
AST (IU/L)	76.15	61.72	51.65	4.95	0.125
AST/ALT	1.93	1.65	1.92	0.15	0.707
TBIL (umol/L)	2.48	2.17	1.95	0.33	0.822
CHOL (mmol/L)	2.26	2.16	2.54	0.09	0.225
TG (mmol/L)	0.28 ^b^	0.40 ^a^	0.34 ^ab^	0.02	0.061
HDL-C (mmol/L)	0.74	0.79	0.82	0.04	0.626
LDL-C (mmol/L)	1.28	1.19	1.44	0.06	0.224
TBA (umol/L)	17.43 ^b^	27.30 ^ab^	30.87 ^a^	2.32	0.038

The values are the means from 6 individual pigs. ^a,b,c^ In the same row, the values with different small superscript letters indicate a significant difference (*p* < 0.05). ^d^ SEM: pooled standard error of the means. C-LT (low-temperature control group), B-LT (basal diet supplemented with full-fat rice Bran group), G-LT (basal diet supplemented with glucose group); for statistical indicator abbreviation, SEM (standard error of the mean); for detection indicator abbreviations, TP (total protein), ALB (albumin), GLB (globulin), ALT (alanine aminotransferase), AST (aspartate aminotransferase), TBIL (total bilirubin), CHOL (total cholesterol), TG (triglyceride), HDL-C (high-density lipoprotein cholesterol), LDL-C (low-density lipoprotein cholesterol), TBA (total bile acid).

## Data Availability

The datasets produced and/or analyzed during the current study are available from the corresponding author on reasonable request. The raw data of the 16S rDNA gene sequencing has been shared in the NCBI databases (https://www.ncbi.nlm.nih.gov/sra/PRJNA1280260 (accessed on 6 August 2025)).

## Supplementary Materials

The following supporting information can be downloaded at: https://www.mdpi.com/article/10.3390/ani15223232/s1, Figure S1: Effects of Dietary Full-Fat Rice Bran or Glucose on the General-Level Abundance of Pig Colonic Microbiota (Non-Prominent Bacterial Genera) During Cold Stress. Data were displayed as the means ± standard error of measurement (SEM), “*” means *p* < 0.05, “**” means *p* < 0.01.
