# Supplementary material for: Dietary Full-Fat Rice Bran or Glucose Regulates Bile Acid Circulation, Colonic Microbiota, and Short-Chain Fatty Acids in Pigs During Chronic Cold Stress
Source: Animals (Basel). 2025 Nov 7;15(22):3232. doi: 10.3390/ani15223232 (PMC12649328; doi:10.3390/ani15223232)
Supplement: Supplementary file 1 [file animals-15-03232-s001.zip › animals-3899514-supplementary.pdf]

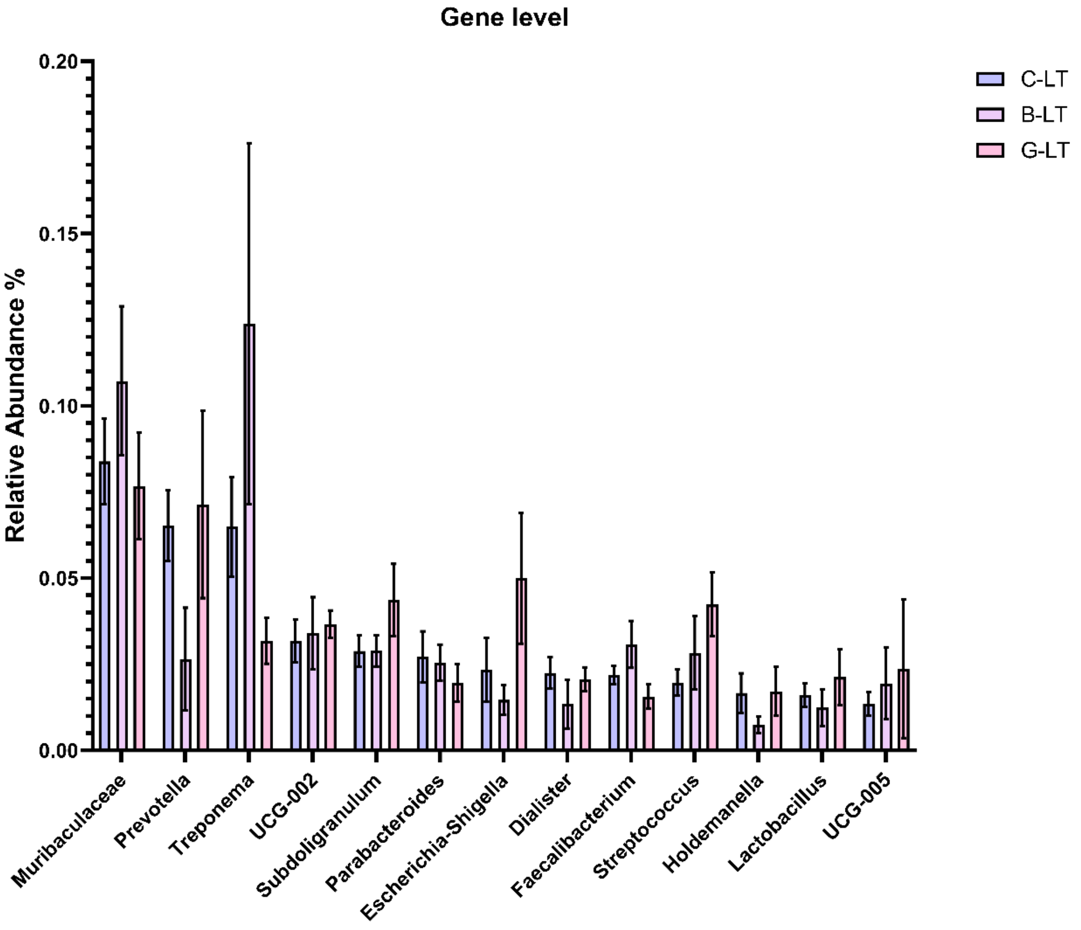

Figure S1. Effects of Dietary Full-Fat Rice Bran or Glucose on the General-Level Abundance of Pig Colonic Microbiota (Non-Prominent Bacterial Genera) During Cold Stress. Data were displayed as the means ± standard error of measurement (SEM), “*” means $P<0.05$, “**” means $P<0.01$.
